# Protocol for the colocalization of yeast peroxisomal membrane proteins and their binding partners using stimulated emission depletion microscopy

**DOI:** 10.1016/j.xpro.2025.103998

**Published:** 2025-08-22

**Authors:** Frank N. Mol, Eline M.F. de Lange, Ida J. van der Klei, Rifka Vlijm

**Affiliations:** 1 Molecular Biophysics, Zernike Institute for Advanced Materials, University of Groningen, Nijenborgh 7, 9747 AG Groningen, the Netherlands; 2 Molecular Cell Biology, Groningen Biomolecular Sciences and Biotechnology Institute, University of Groningen, Nijenborgh 7, 9747 AG Groningen, the Netherlands

**Keywords:** Cell Biology, Cell-based Assays, Microscopy

## Abstract

Peroxisomes are highly dynamic organelles that play crucial roles in cellular metabolism. Here, we present a protocol to investigate peroxisomal membrane proteins and their binding partners in the yeast *Hansenula polymorpha* using stimulated emission depletion (STED) nanoscopy. We describe steps for strain construction to facilitate specific STED-compatible labeling, automated STED imaging, and semi-automated data analysis. This protocol enables examination of the dynamic alterations in the colocalization of the peroxisomal membrane protein Pex3 and its binding partner Atg30.

For complete details on the use and execution of this protocol, please refer to de Lange et al.^1^

## Highlights

• Approach for super-resolution microscopy of yeast peroxisomal membrane proteins
• Steps for constructing yeast strains with specific STED-compatible labeling
• Instructions for acquiring datasets through automated STED imaging
• Guidelines for analyzing STED images of peroxisomal membrane proteins in yeast

## Abstract (editor highlights)

Publisher’s note: Undertaking any experimental protocol requires adherence to local institutional guidelines for laboratory safety and ethics.

## Graphical abstract

**Figure:**
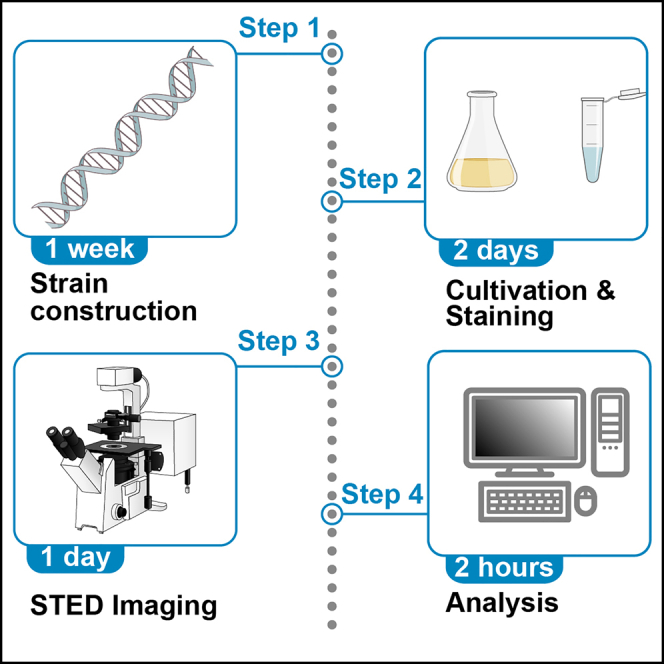

## Before you begin

This protocol facilitates the subcellular localization of binding partners of a peroxisomal membrane protein, as demonstrated by de Lange et al.^1^ This study culminated in a model that describes the repositioning of *Hansenula polymorpha* Atg30, a protein that is involved in pexophagy and which binds to the peroxisomal membrane protein Pex3. Upon induction of pexophagy, Atg30 relocalizes from in between clustered peroxisomes to the exposed part of these organelle clusters. Since diffraction-limited imaging of the peroxisomal membrane lacks the resolution to determine the Pex3 protein distribution, super-resolution microscopy is used in the form of stimulated emission depletion (STED) nanoscopy.^2^^,^^3^^,^^4^ To support comprehensive analysis of the proteins Pex3 and Atg30, large datasets were obtained via automated STED measurements.^5^ A comprehensive semi-automated analysis workflow was developed in Python to obtain quantitative insights into Atg30-Pex3 colocalization, their distribution on peroxisomes and peroxisome clusters, and the spatial arrangement of the observed proteins relative to one another. This protocol aims to guide a user through the entire process, enabling readers to reproduce our study and adapt the protocol for future work, including the steps of strain construction, cell cultivation, imaging, and analysis. Possible applications include colocalization studies of membrane and membrane associated proteins on any spherical cellular organelle.

## Key resources table

**Table:** 

REAGENT or RESOURCE	SOURCE	IDENTIFIER
**Bacterial and virus strains**		

E. *coli* DH5α	Hanahan et al.^6^	N/A

**Chemicals, peptides, and recombinant proteins**		

SiR-Halo, JF585-Halo	Provided by Prof. Dr. Stefan W. Hell (Max Planck Institute for Medical Research, Heidelberg)	N/A
SNAP-Cell 647-SiR	New England Biolabs	Cat#S9102S
FM 4-64 dye (N-(3-triethylammoniumpropyl)-4-(6-(4-(diethylamino) phenyl) hexatrienyl) pyridinium dibromide)	Invitrogen	Cat#T13320
DreamTaq DNA polymerase	Thermo Fisher Scientific	#EP0703
T4 DNA ligase	Thermo Fisher Scientific	#EL0016
Na_2_HPO_4_ · H_2_O	Sigma-Aldrich	7558-79-4
NaCl	Sigma-Aldrich	7647-14-5
KCl	Sigma-Aldrich	7447-40-7
KH_2_PO_4_	Sigma-Aldrich	7758-11-4
Mowiol 4-88 (polyvinylalcohol)	Sigma-Aldrich	81381
Glycerol	Boom	18420000054
Tris	Roche Diagnostics	10708976001
EDTA (Titriplex-III)	Sigma-Aldrich	6381-92-6
DTT	Thermo Fisher Scientific	R0861
MgCl_2_	Sigma-Aldrich	7791-18-6
Zymolyase	Carl Roth	37340-57-1
Sorbitol	Sigma-Aldrich	S1876
Na_2_HPO_4_ · 7H_2_O	Merck	7782-85-6
NaH_2_PO_4_ · H_2_O	Merck	10049-21-5
Peptone	Gibco	2024469
(NH_4_)_2_SO_4_	Sigma-Aldrich	7783-20-2
MgSO_4_ · 7H_2_O	Acros Organics	10034-99-8
Yeast extract	Gibco	2441197
Biotin	Sigma-Aldrich	58-85-5
NaOH	Sigma-Aldrich	1310-73-2
K_2_HPO_4_	Sigma-Aldrich	7758-11-4
KH_2_PO_4_	Sigma-Aldrich	7778-77-0
Thiamine hydrochloride	Sigma-Aldrich	T1270
Riboflavin	Sigma-Aldrich	R9504
Nicotinic acid	Sigma-Aldrich	N4126
p-aminobenzoic acid	Sigma-Aldrich	A9878
Pyridoxal hydrochloride	Carl Roth	Carl Roth
Calcium pantothenate	Sigma-Aldrich	C8731
Inositol	Sigma-Aldrich	I7508
Glucose	Sigma-Aldrich	14431-43-7
Methanol	Sigma-Aldrich	67-56-1
L-leucine	Sigma-Aldrich	L8912

**Critical commercial assays**		

GeneJET PCR purification kit	Thermo Fisher Scientific	#K0701

**Deposited data**		

All raw data are publicly available via DataverseNL	De Lange et al.^1^	DataverseNL: https://doi.org/10.34894/FNUHXH

**Experimental models: Organisms/strains**		

*H. polymorpha yku80*: NCYC495 *YKU80::URA3*, *leu1.1*	Saraya et al.^7^	N/A
*H. polymorpha* P_PEX3_ Pex3-Halo: *yku80* with integration of plasmid pHIPZ_Pex3_Halo; *leu1.1*, Zeo^R^	De Lange et al.^1^	N/A
*H. polymorpha* P_PEX3_ Pex3-GFP: *yku80* with integration of plasmid pSEM61*; leu1.1,* Zeo^R^	De Lange et al.^1^	N/A
*H. polymorpha* P_PEX3_ Pex3-SNAP: *yku80* with integration of plasmid pHIPZ_Pex3_SNAP; *leu1.1*, Zeo^R^	De Lange et al.^1^	N/A
*H. polymorpha* P_PEX3_ Pex3-SNAP: *yku80* with integration of plasmid pHIPN_Pex3_SNAP; *leu1.1*, Nat^R^	De Lange et al.^1^	N/A
*H. polymorpha* P_PEX3_ Pex3-SNAP P_VAC8_ Vac8-Halo: *yku80* Pex3_SNAP with integration of plasmid pHIPZ_Vac8_Halo; *leu1.1*, Zeo^R^, Nat^R^	De Lange et al.^1^	N/A
*H. polymorpha* P_PEX3_ Pex3-SNAP P_ATG30_ Atg30-Halo: *yku80* Pex3_SNAP with integration of plasmid pHIPZ_Atg30_Halo; *leu1.1*, Zeo^R^, Nat^R^	De Lange et al.^1^	N/A
*H. polymorpha* P_PEX3_ Pex3-SNAP P_INP1_ Inp1-Halo: *yku80* Pex3_SNAP with integration of plasmid pHIPZ_Inp1_Halo; *leu1.1*, Zeo^R^, Nat^R^	De Lange et al.^1^	N/A
*H. polymorpha* P_PEX3_ Pex3-SNAP P_PEX19_ Pex19-Halo: *yku80* Pex3_SNAP with integration of plasmid pHIPZ_Pex19_Halo; *leu1.1*, Zeo^R^, Nat^R^	De Lange et al.^1^	N/A
*H. polymorpha pex3* (RBG1): NCYC495 *PEX3::URA3, leu1.1*	Baerends et al.^8^	N/A
*H. polymorpha pex3* P_PEX19_ Pex19-Halo: *pex3* with integration of plasmid pHIPZ_Pex19_Halo; *leu1.1*, Zeo^R^, Nat^R^	De Lange et al.^1^	N/A
*H. polymorpha* P_PEX3_ Pex3-Halo: *yku80* with integration of plasmid pHIPN_Pex3_Halo; *leu1.1*, Nat^R^	De Lange et al.^1^	N/A
*H. polymorpha* P_ATG30_ Atg30-GFP: *yku80* with integration of plasmid pAMK182; *leu1.1*, Zeo^R^	Wu et al.^9^	N/A
*H. polymorpha* P_ATG30_ Atg30-GFP P_PEX3_ Pex3-Halo: *yku80* Atg30_GFP with integration of plasmid pHIPN_Pex3_Halo; *leu1.1*, ZeoR, NatR	De Lange et al.^1^	N/A
*H. polymorpha* P_PEX19_ Pex19-GFP P_PEX3_ Pex3-Halo: *yku80* producing Pex19_mGFP with integration of plasmid pHIPN_Pex3_Halo; *leu1.1*, Zeo^R^, Nat^R^	De Lange et al.^1^	N/A
*H. polymorpha* P_VAC8_ Vac8-GFP P_PEX3_ Pex3-Halo: *yku80* producing Vac8_mGFP with integration of plasmid pHIPN_Pex3_Halo; *leu1.1*, Zeo^R^, Nat^R^	De Lange et al.^1^	N/A
*H. polymorpha* P_INP1_ Inp1-GFP P_PEX3_ Pex3-Halo: *yku80* producing pAMK6 with integration of plasmid pHIPN_Pex3_Halo; *leu1.1*, Zeo^R^, Nat^R^	De Lange et al.^1^	N/A

**Oligonucleotides**		

Primer Ppex3_Fw: CCTGTTGCGGCAAGATATAG	De Lange et al.^1^	N/A
Primer Halo_Rv: CTTGCAGCAGATTCAGACC	De Lange et al.^1^	N/A
Primer Snap_Rv: GAATGGGCACGGGATTTC	De Lange et al.^1^	N/A
Primer Pex19_Fw: CCATGTTTAAGCTTATGAGCGAG	De Lange et al.^1^	N/A
Primer Pex19_BamHI_Rv: GCACGCGGATCCTGTT TGTTTGCAAGTGTCTTC	De Lange et al.^1^	N/A
Primer Ppex19_Fw: CTGCTCGTCTATCTATTTAGGC	De Lange et al.^1^	N/A
Primer Pinp1_Fw: GCTCATCGCTTATGTCACC	De Lange et al.^1^	N/A
Primer Patg30_Fw: GACTTAGCACGCCTTGGCTC	De Lange et al.^1^	N/A
Primer Pvac8_Fw: GGCTACCCAGGATAAGAAC	De Lange et al.^1^	N/A

**Recombinant DNA**		

Plasmid pUC57_Halo: pUC57 plasmid containing 45bp Halo linker and HaloTag7 fragment; Amp^R^	GenScript	N/A
Plasmid pUC57_SNAP: pUC57 plasmid containing pSNAPf-tag fragment; Amp^R^	GenScript	N/A
Plasmid pSEM61: pHIPZ encoding the C-terminal part of Pex3 fused to mGFP; Zeo^R^, Amp^R^	Wu et al.^10^	N/A
Plasmid pHIPZ_Pex3_Halo: pHIPZ containing the C-terminal part of Pex3 fused to HaloTag; Zeo^R^, Amp^R^	De Lange et al.^1^	N/A
Plasmid pHIPN_Pex3_Halo: pHIPN containing the C-terminal part of Pex3 fused to HaloTag; Nat^R^, Amp^R^	De Lange et al.^1^	N/A
Plasmid pHIPZ_Pex3_SNAP: pHIPZ containing the C-terminal part of Pex3 fused to SNAP-tag; Zeo^R^, Amp^R^	De Lange et al.^1^	N/A
Plasmid pHIPN_Pex3_SNAP: pHIPN containing the C-terminal part of Pex3 fused to SNAP-tag; Nat^R^, Amp^R^	De Lange et al.^1^	N/A
Plasmid pAMK6: pHIPZ containing the C-terminal part of Inp1 fused to mGFP; Zeo^R^, Amp^R^	Krikken et al.^11^	N/A
Plasmid pHIPZ_Inp1_Halo: pHIPZ containing the C-terminal part of Inp1 fused to HaloTag; Zeo^R^, Amp^R^	De Lange et al.^1^	N/A
Plasmid pHIPZ_Vac8_mGFP: pHIPZ containing the C-terminal part of Vac8 fused to mGFP; Zeo^R^, Amp^R^	Singh et al.^12^	N/A
Plasmid pAMK174: pHIPZ containing the C-terminal part of Vac8 fused to mKate2; Zeo^R^, Amp^R^	Wu et al.^13^	N/A
Plasmid pAMK178: pHIPN containing the C-terminal part of Vac8 fused to mKate2; Nat^R^, Amp^R^	Wu et al.^13^	N/A
Plasmid pHIPZ_Vac8_Halo: pHIPZ containing the C-terminal part of Vac8 fused to HaloTag; Zeo^R^, Amp^R^	De Lange et al.^1^	N/A
Plasmid pHIPZ_Pex19_mGFP: pHIPZ containing the C-terminal part of Pex19 fused to mGFP; Zeo^R^, Amp^R^	De Lange et al.^1^	N/A
Plasmid pHIPZ_Pex19_Halo: pHIPZ containing the C-terminal part of Pex19 fused to HaloTag; Zeo^R^, Amp^R^	De Lange et al.^1^	N/A
Plasmid pAMK182: pHIPZ containing the C-terminal part of Atg30 fused to mGFP; Zeo^R^, Amp^R^	Wu et al.^9^	N/A
Plasmid pHIPZ_Atg30_Halo: pHIPZ containing the C-terminal part of Atg30 fused to HaloTag; Zeo^R^, Amp^R^	De Lange et al.^1^	N/A

**Software and algorithms**		

Fiji software (ImageJ 2.14.0)	Schindelin et al.^14^	https://imagej.net/software/fiji/; RRID:SCR_002285
Original code	This study	https://doi.org/10.5281/zenodo.11047200
Python version 3.8.8	Python Software Foundation	https://www.python.org; RRID:SCR_008394
Jupyter Notebook version 6.5	Project Jupyter	https://jupyter.org/

**Other**		

Chamlide magnetic imaging chamber for 18 mm round coverslips	Live Cell Instrument Co., Ltd.	CM-B18-1
Class II biological safety cabinet or a flame	N/A	N/A
Clean borosilicate glass coverslips (18 mm, No. 1.5H)	VWR	CAT#631-1580
Electroporator	BTX	ECM600
Glass microscope slides	Epredia	AG00008432E01MNZ20
Heating block at 37°C or 65°C	AccuBlock digital dry bath	Labnet
Magnetic hotplate stirrer at 50°C	VWR	442-1270
Milli-Q ultrapure water purification system	Merck	Z00QSVC01
NanoDrop One	Thermo Fisher Scientific	ND-ONE-W
Innova 44 incubator shaker	New Brunswick	M1282-0002
Spectrophotometer	StaRRcol	SC-60-S
Tabletop centrifuge	Eppendorf	5425
Vortex Genius 3	IKA	0003340000

## Materials and equipment

### Reagents

**Table:** PBS 10×

Reagent	Final concentration	Amount
Na_2_HPO_4_	100 mM	1.44 g
NaCl	1.37 M	8 g
KCl	27 mM	0.20 g
KH_2_PO_4_	18 mM	0.24 g
dH_2_O	N/A	Bring to 100 mL
**Total**	**N/A**	**100 mL**

Sterilize by autoclaving. Store the 10× (1 M) stock at 19°C–22°C for up to 12 months. When diluted to 1× PBS (0.1 M), adjust the pH to 7.4 with 1 M NaOH.

**Table:** Mowiol

Reagent	Final concentration	Amount
Mowiol 4-88 (Polyvinylalcohol)	10% w/v	10 g
Glycerol	25% w/v	25 g
Tris	50 mM	0.6 g
dH_2_O	N/A	Bring to 100 mL
**Total**	**N/A**	**100 mL**

Adjust the pH to 8.5 with 1 M HCl. Slowly stir for at least 24 hours at 50°C to dissolve the Mowiol. Centrifuge at 5000 *g* for 15 minutes to remove any undissolved solids. Store 1 mL aliquots at −20°C for up to 12 months. Warm tubes to 19°C–22°C for use. Opened tubes can be stored at 4°C for 1 month.

**Table:** TED

Reagent	Final concentration	Amount
Tris	100 mM	1.20 g
EDTA	50 mM	1.48 g
DTT	25 mM	0.39 g
dH_2_O	N/A	Bring to 100 mL
**Total**	**N/A**	**100 mL**

Adjust the pH to 8.0 with 1 M HCl. Sterilize by autoclaving and add DTT right before use. Store at 19°C–22°C for up to 12 months.

**Table:** STM

Reagent	Final concentration	Amount
Sucrose	270 mM	9.24 g
Tris	10 mM	120 mg
MgCl_2_	1 mM	9.52 mg
dH_2_O	N/A	Bring to 100 mL
**Total**	**N/A**	**100 mL**

Adjust the pH to 8.0 with 1 M HCl. Sterilize by autoclaving and store at 19°C–22°C for up to 12 months.

**Table:** Zymolyase Solution

Reagent	Final concentration	Amount
Zymolyase (ICN)	2.5 mg/mL	250 mg
Sorbitol	1.2 M	21.86 mg
Na_2_HPO_4_ · 7H_2_O	75 mM	2.02 mg
NaH_2_PO_4_ · H_2_O	25 mM	339 mg
dH_2_O	N/A	Bring to 100 mL
**Total**	**N/A**	**100 mL**

Add the Zymolyase and sorbitol to the phosphate buffer (100 mM, pH 7.4).

Store aliquots at −20°C for at least 6 months. Make aliquots of a maximum of 100 μL and do not freeze-thaw the solution more than twice.

**Table:** YPD

Reagent	Final concentration	Amount
Yeast Extract	1% w/v	1 g
Peptone	1% w/v	1 g
Glucose	1% w/v	1 g
dH_2_O	N/A	Bring to 100 mL
**Total**	**N/A**	**100 mL**

Sterilize by autoclaving and store at 19°C–22°C for up to 12 months. For plates, 2% w/v agar (2 g) can be added before sterilizing.

**Table:** Mineral medium for *H. polymorpha* (van Dijken et al.^15^)

Reagent	Final concentration	Amount
(NH_4_)_2_SO_4_	19 mM	250 mg
MgSO_4_ · 7H_2_O	0.8 mM	20 mg
K_2_HPO_4_	4 mM	70 mg
NaH_2_PO_4_	25 mM	300 mg
yeast extract	0.05% w/v	50 mg
Vishniac solution (1000×)	0.1% v/v	0.1 mL
Vitamin solution (1000×)∗	0.1% v/v	0.1 mL
dH_2_O	N/A	Bring to 100 mL
**Total**	**N/A**	**100 mL**

When (NH_4_)_2_SO_4_ is omitted, add 1 g/L K_2_SO_4_. Sterilize by autoclaving. ∗Add vitamin solution after autoclaving the medium. Store at 19°C–22°C for up to 12 months.

**Table:** Vitamin Solution 1000×

Reagent	Final concentration	Amount
Biotin	0.01% w/v	10 mg
NaOH	0.1 M	10 mL
K_2_HPO_4_	7.36 mM	128 mg
KH_2_PO_4_	2.64 mM	36 mg
Thiamine	0.02% w/v	20 mg
Riboflavin	0.01% w/v	10 mg
Nicotinic acid	0.5% w/v	500 mg
p-Aminobenzoic Acid	0.03% w/v	30 g
Pyridoxal hydrochloride	0.01% w/v	10 mg
Ca-pantothenate	0.2% w/v	200 mg
Inositol	1% w/v	1 g
dH_2_O	N/A	Bring to 100 mL
**Total**	**N/A**	**100 mL**

Dissolve the biotin in 0.1 M NaOH. Add the potassium phosphate buffer (10 mM, pH 7.5). Then dissolve the other compounds. Sterilize the solution by filtration through a 0.2 μm filter. Store at 4°C for up to 6 months. Add to mineral medium after autoclaving.

**Table:** Vishniac solution 1000× (Vishniac et al.^16^)

Reagent	Final concentration	Amount
EDTA (Titriplex-III)	27 mM	1 g
ZnSO_4_.7H_2_O	15 mM	440 mg
MnCl_2_.4H_2_O	5.1 mM	101 mg
CoCl_2_.6H_2_O	2.5 mM	32 mg
CuSO_4_.5H_2_O	1.3 mM	31.5 mg
(NH_4_)_6_Mo_7_O_24_.4H_2_O	0.2 mM	22 mg
CaCl_2_.2H_2_O	10 mM	147 mg
FeSO_4_.7H_2_O	3.6 mM	100 mg
dH_2_O	N/A	Bring to 100 mL
**Total**	**N/A**	**100 mL**

Dissolve first EDTA and subsequently ZnSO_4_ in 50 mL dH_2_O. Adjust the pH to 6 with 10 M NaOH. Dissolve the other components one by one and adjust the pH each time to 6. Finally, adjust the pH to 4.0 with 1 M HCl. Sterilize the Vishniac solution by autoclaving. Store at 4°C in the dark for up to 12 months. Initially, the solution has a green color, later it turns purple. Add to mineral medium.

**Table:** Carbon source 100×

Reagent	Final concentration	Amount
Glucose	50% (w/v)	50 g
Methanol	50% (v/v)	50 mL
dH_2_O	N/A	Bring to 100 mL
**Total**	**N/A**	**100 mL**

The stock solution, either glucose or methanol, is diluted 100× in the medium to a 1× solution (0.5%). Sterilize by autoclaving (glucose solution) or by filtration through a 0.2 μm filter and store at 19°C–22°C for up to 12 months.

**Table:** Additional constituent: Leucine 100×

Reagent	Final concentration	Amount
L-leucine	6 mg/mL	600 mg
dH_2_O	N/A	Bring to 100 mL
**Total**	**N/A**	**100 mL**

The stock solution is diluted 100× in the medium to a 1× solution (60 mg/L). Sterilize by autoclaving and store at 19°C–22°C for up to 12 months.

**1% Formaldehyde (FA):** Incubate 16% (w/v) FA (stored at −20°C for up to 12 months) at 65°C on a heat block. Dilute 16× in 1× PBS to make a 1% (w/v) FA solution (62.5 μL / 1 mL PBS).

### Microscope and software

#### STED microscope

This protocol is compatible with standard STED microscopes equipped with 488 nm and 640 nm excitation lasers combined with a 775 nm depletion laser, and using a 100×/1.4 NA oil immersion objective. To make use of the automated workflow, microscope control is needed through Python. Our study was performed using the Abberior Expert Line (https://abberior.rocks/) and the SpecPy Python package (https://imspectordocs.readthedocs.io/en/latest/specpy.html). In the case of live-cell imaging, the microscope must be preheated and kept at 37°C.

#### Python

Python is an open-source programming language, which is used for automated STED measurements and to perform the semi-automated analysis of the data. The version needs to be 3.8.8 or above. To use Python, a distribution can be downloaded from the Python website (https://www.python.org/).

#### Jupyter Notebook

Jupyter Notebook is an open-source program for interactive Python scripts, that is used for the STED automation and the data analysis. Install the Jupyter Notebook environment through the Python installation environment: *pip install jupyter notebook*, or visit the Jupyter website (https://jupyter.org/).

#### STED automation

To run the automation, see Mol et al.^5^ This automation is written in Python (>= 3.8.8).

#### Automated analysis

The analysis workflow is written in Python (>= 3.8.8) and can be obtained from the open-source repository^17^ (https://doi.org/10.5281/zenodo.11047200).

## Step-by-step method details

### Construction of *H. polymorpha* strains

**Timing: 1 week**

This step describes the transformation of plasmid DNA in the yeast *H. polymorpha*. Yeast strains are constructed for tagging the proteins of interest, e.g., HaloTag for Pex3 and green fluorescent protein (GFP) for Atg30.

1. Grow the *H. polymorpha* cells for 12–20 h at 37°C in YPD medium until they reach the stationary growth phase.
2. Inoculate 1 mL of the culture into 100 mL (prewarmed) YPD medium and grow the cells at 37°C to an OD_600_ of 1.2–1.5 (this will take approximately 6 h, measured using the StaRRcol SC-60-S spectrophotometer).
3. Centrifuge the 100 mL culture for 5 min at 1800 × *g* at 19°C–22°C and discard the supernatant.
4. Resuspend the cells in 25 mL TED. TED is a Tris-buffered solution containing EDTA to bind metal ions and DTT to reduce protein disulfide bonds. These components enhance the transformation efficiency.
5. Incubate the cells for 15 min at 37°C with shaking (200 rpm).
6. Centrifuge the cells for 5 min at 1800 × *g* at 19°C–22°C and discard the supernatant.
7. Wash the cells by resuspending the pellet in 100 mL ice-cold STM buffer. The cells need to remain cold from this point on, to keep the cell membranes permeable and ensure efficient uptake of the plasmid DNA. At low temperatures, lipids move less, so they cannot seal the holes made by the procedure to make the cells competent.
8. Centrifuge the solution for 5 min at 1800 × *g* at 4°C and discard the supernatant.
9. Wash the cells by resuspending them in 50 mL ice-cold STM.
10. Centrifuge the cells for 5 min at 1800 × *g* at 4°C and discard the supernatant.
11. Resuspend the cells in 0.5 mL ice-cold STM.
12. Divide the cell suspension in batches of 60 μL and:
  a. Use immediately, or
  b. Freeze in liquid N_2_ and store at −80°C.
13. Add the plasmid DNA (max. 3–4 μL, ≈1 μg).

***Note:*** Linearize the plasmid with the DNA of interest (for example Atg30-Halo) for integration into the genome.

14. Transfer the cell-DNA mixture into a 2 mm electroporation cuvette (PN620).
  a. Pulse settings:
    i. 50 μF.
    ii. 129 Ohm.
    iii. 1.5 kV (7.5 kV/cm).
    iv. Electro-pulse - resulting pulse length will be 4–5 ms.
    v. Immediately add 940 μL YPD (at 19°C–22°C) to the cell-DNA mixture and transfer the suspension to a 2 mL microcentrifuge tube.
15. Incubate the cells for 1 h at 37°C with shaking (200 rpm).
16. Plate 10% of the cells on selective YPD plates (with appropriate antibiotics).
17. Centrifuge the remaining 900 μL of cells for 1 min at 1800 × *g* at 19°C–22°C to concentrate and plate on selective YPD plates.
18. Colonies appear after 1.5 days on selective YPD plates.

***Note:*** The selective plates must contain the proper antibiotics. **(2)** This will yield >10^6^ transformants per μg plasmid DNA. Obtained from Faber et al.^18^

19. Take a colony from one of the fresh plates and resuspend it in 10 μL of Zymolyase solution.

***Note:*** Zymolyase is an enzyme that is used to degrade the cell wall of yeast cells, in order to form spheroplasts (see step 14).

20. Incubate the solution for 15 min at 37°C in a heating block (for the formation of spheroplasts).

***Note:*** Spheroplasts are yeast cells with their cell wall largely removed. This helps to extract the DNA from these cells, needed for PCR. Spheroplast formation can be visualized using a light microscope, to check if the cell wall has disappeared.

21. Add 20 μL of ultrapure water and resuspend the spheroplasts. Spheroplasts can be stored up to 1 month at −80°C.
22. Use 1 μL of the spheroplast suspension for performing the colony PCR.
23. Perform the colony PCR in 20 μL final volume and use DreamTaq enzyme to amplify the desired product.
24. Load 10 μL of the PCR product on an agarose gel.

***Note:*** If possible also add a positive and negative control during PCR.

### Cultivation and staining

**Timing: 2 days**

This part of the protocol describes sample preparation by fluorescent labeling of the genetically modified cells of the *H. polymorpha* strains. This part is divided into live cell and fixed cell sample preparation. Follow the appropriate steps accordingly.

25. Grow the cells for 12–20 h at 37°C in a flask, shaking (200 rpm, until in the stationary phase) in mineral medium (MM) supplemented with 0.5% (w/v) glucose.
26. Dilute the culture to OD_600_=0.1 in MM supplemented with 0.5% (w/v) glucose and grow the cells until an OD_600_≈1.6 (this will take approximately 5 h).
27. Dilute the culture again to OD_600_=0.1 in MM supplemented with 0.5% (v/v) methanol and grow the cells for 4–16 h at 37°C shaking.

***Note:*** Growth time on methanol is dependent on the desired size of the peroxisomes, as shown in de Lange et al.^1^

28. Harvest 5 OD_600_ units of cells by centrifuging for 1 min at 1800 × *g* at 19°C–22°C (Eppendorf tabletop centrifuge 4525).

***Note:*** For staining, follow either step 29 or step 30, depending on whether live cell imaging or fix cell imaging is performed, respectively.

29. Option 1: Sample preparation for live cell imaging.
  a. Stain the cells by resuspending them in a solution of 1 μM dye in 500 μL complete MM (with carbon source).
  b. Incubate the cells for 1 h at 37°C shaking (in the dark).
    ***Note:*** SiR-Halo, JF585-Halo, or SNAP-Cell 647-SiR dyes can for example be used.
  c. Centrifuge the cells for 1 min at 1800 × *g* at 19°C–22°C and discard the supernatant.
  d. Wash the cells once with 1 mL MM without dye and centrifuge again as above (1 min, 1800 × *g* at 19°C–22°C).
  e. Resuspend the cells in 500 μL MM without dye.
  f. Add the suspension to the live-cell imaging chamber.
  g. Transfer the imaging chamber to the STED microscope, which is preheated and kept at 37°C.
30. Option 2: Sample preparation for fixed cell imaging.
  a. Wash the cell pellet with 2 mL cold PBS (pH 7.4).
  b. Centrifuge the cells for 1 min at 1800 × *g* at 19°C–22°C and discard the supernatant.
  c. Resuspend the cell pellet in 500 μL cold 2% (w/v) FA in PBS and keep for 30 min on ice.
  d. Coat ethanol-washed glass coverslips with Poly-L-lysine (30 min at 19°C–22°C) in a 12-well plate, rinse three times with PBS, and air dry.
  e. Centrifuge the cells for 1 min at 1800 × *g* at 19°C–22°C and discard the supernatant.
  f. Wash the cells once with 2 mL PBS.
  g. Centrifuge the cells for 1 min at 1800 × *g* at 19°C–22°C and discard the supernatant.
  h. Stain the cells by resuspending the cell pellet in a solution of 1 μM dye in 500 μL cold PBS.
  i. Incubate the cells for 1 h at 37°C shaking (in the dark).
  j. Centrifuge the cells for 1 min at 1800 × *g* at 19°C–22°C and discard the supernatant.
  k. Wash the cells with 2 mL PBS, centrifuge for 1 min at 1800 × *g* at 19°C–22°C and discard the supernatant. Repeat this step two times.
  l. Resuspend the cells in 200 μL PBS and pipette the suspension on a coated coverslip in a well of the 12-well plate.
  m. Incubate the cells for 15 min at 37°C (in the dark).
  n. Discard the solution and wash the coverslip that contains the cells once with PBS.
  o. Mount the coverslip using Mowiol on a glass microscope slide (invert the coverslip on it).
  p. Let the sample dry for 12–20 h in the dark.

**Pause point:** Store the slide at 4°C in the dark until use or transfer directly to the STED microscope.

### STED imaging

**Timing: 1 day**

This step describes the automated STED imaging, including the setup of the automation and the imaging parameters.

**Figure 1:**
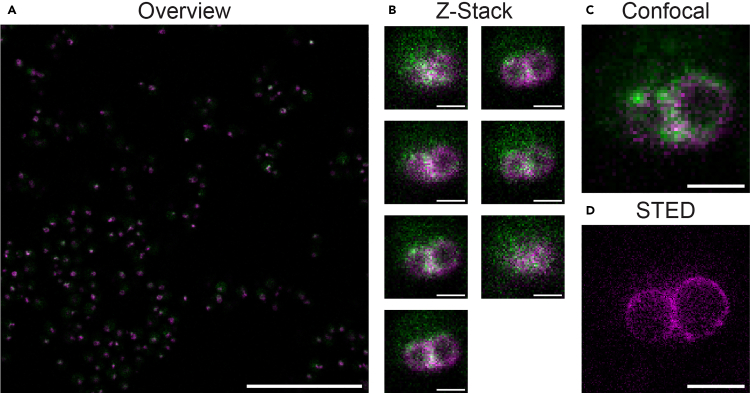
Data acquisition: Examples of the confocal and STED images of the peroxisomal membrane protein Pex3 (magenta) and its binding partner Atg30 (green) in the yeast *Hansenula polymorpha* (A) Overview image to visualize both proteins and localize individual yeast cells. Scale bar, 25 μm. (B) Confocal z-stack to provide three-dimensional context of the resulting STED and confocal image, and to determine the optimal focus position based on the Pex3 fluorophore distribution in each slice. Scale bar, 1 μm. (C) Image with optimal confocal settings for the binding partner Atg30. A confocal image with low-resolution settings is measured for Pex3 as a control. Scale bar, 1 μm. (D) STED image of the peroxisomal membrane protein Pex3. A confocal image with low-resolution settings is measured for Atg30 as a control. Scale bar, 1 μm.
  b. At the position of each selected yeast cell, a confocal z-stack is acquired to provide 3-dimensional context and to determine the correct focus position, based on the Pex3 label (Figure 1B). This z-stack is obtained using mid-resolution confocal imaging settings, e.g.:
    i. Pixel size (60 nm).
    ii. FOV (3 × 3 μm^2^, adjust the FOV size depending on the cell size. Use width and height of approximately two times the diameter of the cell).
    iii. Stack (7 slices with 200 nm spacing).
    iv. Laser settings (0.10% 640 nm, 10.0% 488 nm).
    v. Detector spectral range (650–757 nm, 495–550 nm).
    vi. Pixel dwell time (20.0 μs).
    vii. Line steps (1 for the 640 nm channel, 1 for the 488 nm channel).
    viii. Line accumulations (2).
    ix. Pinhole (1.25 AU).
  c. For each selected cell, a confocal image is acquired to obtain detailed information on the protein (Atg30) labeled with GFP. The optimal focus position, as determined in the previous step, is used (Figure 1C). Perform this confocal measurement in the 488 nm channel, use high-resolution confocal imaging settings, e.g.:
    i. Pixel size (60 nm).
    ii. FOV (3 × 3 μm^2^, adjust the FOV size depending on cell size. Use width and height of approximately two times the diameter of the cell).
    iii. Laser settings (0.10% 640 nm, 10.0% 488 nm).
    iv. Detector spectral range (650–757 nm, 495–550 nm).
    v. Pixel dwell time (20.0 μs).
    vi. Line steps (1 for the 640 nm channel, 20 for the 488 nm channel).
    vii. Line accumulations (1).
    viii. Pinhole (0.70 AU).
  d. The high-resolution STED measurement provides precise localization of the Pex3 protein, for each cell at the optimal focus position (Figure 1D). This STED measurement is done in the 640 nm channel, use high-resolution STED settings, e.g.:
    i. Pixel size (20 nm).
    ii. FOV (3 × 3 μm^2^, adjust the FOV size depending on cell size. Use width and height of approximately two times the diameter of the cell).
    iii. Laser settings (1.0% 640 nm combined with 22.0% 775 nm).
    iv. Detector spectral range (650–757 nm).
    v. Pixel dwell time (10.0 μs).
    vi. Line steps (12 for the 640 nm channel).
    vii. Line accumulations (2).
    viii. Pinhole (0.80 AU).

***Note:*** Suggested settings are provided in parentheses. For optimal setup and sample-dependent settings, optimize all channels.

***Note:*** In step 32c, a low-resolution image of the peroxisomal membrane protein in the alternate channel is acquired as a control. Since 32c and 32d are measured consecutively, potential drift can be detected by comparing the positions of the measured structure in the control image with the final STED image.

31. Set up the four different imaging configurations on the STED microscope:
  a. Set up a configuration for overview measurements using confocal microscopy, that measures the labeled proteins simultaneously (480 nm and 640 nm excitation) in a large FOV (e.g., 80 × 80 μm^2^).
  b. Set up a configuration for confocal z-stack measurements for measuring the 480 nm and 640 nm channels simultaneously, to obtain a three-dimensional context for each cell separately. Set the lateral dimensions to 3 × 3 μm^2^ (width and height of approximately two times the diameter of the cell line used), and span an axial length of 2 μm.
  c. Create a configuration for two-dimensional confocal measurements of the 480 nm channel.
  d. Create a configuration for two-dimensional STED measurements of the 640 nm channel.
32. Optimize all four imaging configurations:
  a. A confocal overview image is measured to determine the positions of yeast cells based on the positions of the Pex3 label in a large FOV (Figure 1A). Low-resolution confocal imaging settings are used, e.g.:
    i. Pixel size (150 nm).
    ii. FOV (80 × 80 μm^2^).
    iii. Laser settings (0.20% 640 nm, 5.0% 488 nm).
    iv. Detector spectral range (650–757 nm, 495–550 nm).
    v. Pixel dwell time (20.0 μs).
    vi. Line steps (1 for the 640 nm channel, 1 for the 488 nm channel).
    vii. Line accumulations (1).
    viii. Pinhole (1.25 AU).

33. Set up the STED automation software:
  a. Open the automation Python software using Jupyter Notebook on the STED microscope computer.
  b. Link the above-defined configurations in the automation software by entering the correct configuration names in the script.
  c. Test and set the threshold parameter (e.g. 30 counts), such that the desired segmentation is performed. A majority of the present cells should be segmented by the software.

***Note:*** The correct segmentation threshold is dependent on the fluorescent signal of the sample and should be tested (and optimized if needed) for each sample to measure.

34. Perform a small test run of the complete automation workflow (e.g. measuring three cells).

**CRITICAL:** Ensure measurement configurations and automation are set up correctly before running the automation on a larger batch.

35. Run the automated STED workflow. Aim for at least 100 cells per sample.

### Analysis

**Timing: 2 h**

**Figure 2:**
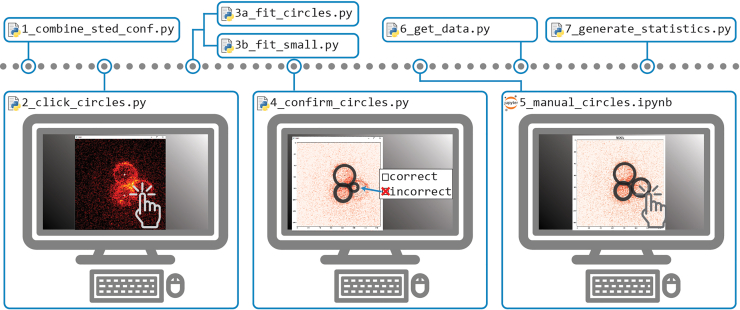
Key steps of the semi-automated analysis workflow using the raw STED and confocal data of the peroxisomal membrane protein and the binding partner These steps enable automated circle fitting for the imaged peroxisomal membrane proteins, with manual control and adjustment of the generated data. This analysis results in statistics on the distribution of the peroxisomal protein across the membrane, the correlation and colocalization of the binding partner, and the corresponding positioning of the binding partner to a cluster of peroxisomes.

This step describes the process for obtaining quantitative insights into the colocalization, protein distribution, and the spatial arrangement of the observed proteins relative to one another, using a semi-automated workflow in Python (Figure 2).

36. Open the semi-automated analysis directory.^17^
37. Transfer the .tiff files of the measured confocal and STED data to the following data directory:

> STED_Pex3_Atg30-Pex3_colocalization/data/sted/

38. Merge the STED and the confocal images into a single stack. The measured 480 nm confocal channel and the 640 nm STED channel must be combined in a single stack. Run:

> 1_combine_sted_conf.py

39. Select the circles in the measured data:
  a. Run:
    > 2_click_circles.py
  b. The script opens all measured stacks (step 38) consequently.
  c. For each circle in the image to be analyzed, select its center by clicking near the midpoint with the left mouse button.
  d. If repositioning of the centers is necessary, right-click to reset.
  e. Press any key to continue to the next image in the dataset.
  f. Repeat until all images have been displayed (no new image will open).
40. Fit the circles automatically. Depending on the size of the circles present in the sample, either run:

**Figure 3:**
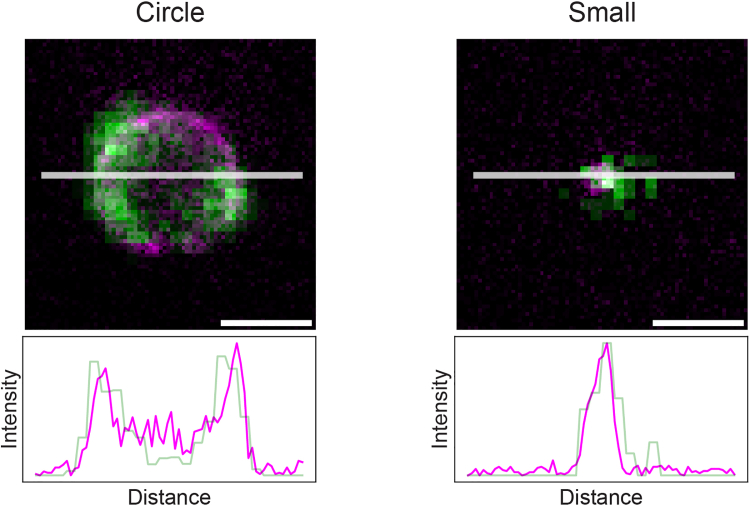
Comparison of fitting methods for circles and small peroxisomes (step 40) The peroxisomal membrane protein Pex3-Halo was labeled with SiR-Halo in *Hansenula polymorpha* cells for both panels. The left panel shows Pex3 in yeast grown in methanol-containing medium for 6 hours. A line profile through the center of the circle results in a plot with two peaks, requiring the use of the script *3a_fit_circles.py*. The right panel shows Pex3 in yeast grown in glucose-containing medium for 6 hours. A line profile through the small peroxisome results in a plot with a single peak, and therefore requires the use of the script *3b_fit_small.py*. Scale bar, 500 nm.

> 3a_fit_circles.py

Or

> 3b_fit_small.py

***Note:*** Use the *3a_fit_circles.py* script if a line profile through the center of the peroxisome shows two peaks, and run the *3b_fit_small.py* script if this line profile shows a single peak (Figure 3). The latter is mainly used for small tagged peroxisomes (Pex3-Halo) in glucose-grown cells.

41. Verify and confirm the automated fitted circles.
  a. Run:
    > 4_confirm_circles.py
  b. All measured data will open consequently.
    i. If the fitted circles are correct, press any key to continue.
    ii. If the fitted circles are incorrect, left-click and press any key to continue.
42. Manually adjust incorrect circles.
  a. Open the following notebook script notebook script in Jupyter Notebook.
    > 5_manual_circles.ipynb
  b. Consequently, all images that were labeled as incorrect (in step 41b) will be presented for adjustments.
    i. Decide whether to exclude the measured image or manually fit the rings by following the commands in the interactive tool.
    ii. If manually fitting is selected, use the tool to click the center of each ring, followed by clicking the perimeter of the ring.
43. Retrieve line profiles. To acquire all line profiles of the annotated rings (annotated, confirmed, and adjusted in steps 40 to 42) of both channels of the image stack, run:

> 6_get_data.py

44. Retrieve all statistics for all stacks by analyzing the acquired line profile data (step 43). Run:

> 7_generate_statistics.py

## Expected outcomes

The expected outcome after following the complete protocol, is insight into the localization of the peroxisomal membrane protein Pex3 and the co-localization with the binding partner Atg30. This follows from the acquired quantifications of Atg30-Pex3 colocalization, their distribution on peroxisomes and peroxisome clusters, and the spatial arrangement of the observed proteins relative to one another. These quantifications are facilitated by the extensive, automatically acquired dataset of super-resolution STED images with ∼30 nm resolution of Pex3 in *H. polymorpha.*

## Quantification and statistical analysis

To determine statistical significance, the resulting quantifications can be grouped and tested by comparing two or more subsets of the data. For example, a t-test can be performed to test for statistically significant differences between two sets with different growth time points. For a significance test involving more than two subsets, use a two-way ANOVA test.

## Limitations

Automated imaging significantly increases the amount of data acquired. As manual data analysis becomes very time-consuming for large amounts of data, here we provided a protocol for automated ring-fitting to enable quantification with minimal manual work. An important limitation of the automated ring-fitting process is that its performance strongly depends on the image quality. Hence, to minimize manual verification and correction, it is important to optimize both the sample preparation (e.g. to ensure optimal labeling density and minimal background fluorescence) and the image acquisition (e.g. optimal signal-to-noise ratio). Also, the number of organelles present in the image could complicate automated analysis (i.e. superimposed rings are harder to fit automatically). However, incorrectly fitted rings can be corrected manually by following our manual-fitting workflow.

Automated imaging itself could increase the incidence of incorrect images as compared to manual imaging (e.g. out-of-focus, incorrect area selection without peroxisome present). These images should be excluded from the analysis, for which a simple routine is provided (steps 41 and 42).

To obtain reliable quantifications, the data set size must be sufficiently large. In the here described work, we aimed to obtain images from at least 100 cells, measured in biological triplicates for each condition. Depending on the structure of interest, possibly even larger datasets are required.

The protocol describes STED imaging of a peroxisomal membrane protein combined with diffraction-limited confocal imaging of its binding partner. The limitation of this approach is that the second protein is localized at a lower precision. To enable localization at the same high resolution for both proteins, dual-color STED imaging could be performed. For dual color labeling with STED dyes, orthogonal labeling is required,^19^ e.g. use the SNAP-tag2^20^ alongside the HaloTag. Dual-color STED analysis can be applied using the same semi-automated workflow as described in this protocol, when omitting step 38 (the step to combine STED and confocal data in one stack). Furthermore, to minimize photobleaching induced by the depletion beam during dual-color imaging, a single depletion wavelength should be used. Accordingly, red and far-red dyes are selected in combination with a 775 nm depletion laser. The additional benefit of using a single depletion laser is the lack of chromatic aberration, omitting additional alignment steps while obtaining very precise information on the (co)localization.

## Troubleshooting

### Problem 1

There is a shift between the positioning of the membrane protein and the secondary label targeting the binding partner. (Imaging).

### Potential solution

Perform alignment of both excitation beams and the STED depletion beam using e.g. Invitrogen Tetraspec 0.1 μm bead samples (Cat. No T7279).

### Problem 2

There is a horizontal shift in the image. (Imaging).

### Potential solution

This is likely caused by drift of the cells or the slide during imaging, which is especially prevalent in live-cell imaging. If this occurs, exclude the data for analysis. Ensure good coating with Poly-L-lysine of the coverslip for correctly adhered cells. If necessary, decrease the dwell time (increase temporal resolution), decrease the number of line repeats, or both (see for more details de Lange et al.,^19^ Figure 3). For fixed slides, ensure the slides are at 19°C–22°C before imaging.

### Problem 3

Faint structures, high background intensity, significant autofluorescence, or a combination of these. (Imaging).

### Potential solution

These problems can have various origins. One cause of faint images is the degradation of fluorophores (especially the Halo-dyes) over time in the (chemical) environment of the sample. To minimize this effect, decrease the time between labeling and imaging the sample. Hence, it is advisable to acquire data on the samples in the same week as preparing them (and store the samples at 4°C). Secondly, over- and under-labeling and background fluorescence can also significantly decrease the signal-to-noise ratio. Make sure that the labeling concentration is optimized and that the washing steps are performed properly. Furthermore, suboptimal (STED) laser powers can also result in decreased signal-to-noise ratio, lowered resolution, increased autofluorescence, heightened background signal, or a combination of these. Therefore, optimize excitation and STED laser powers, as well as the other microscope settings. For further detailed guidelines for optimizing STED protocols for peroxisomal proteins, we refer to de Lange et al.^19^

### Problem 4

There is a mismatch between the automation software and the microscopy software. (Imaging).

### Potential solution

Ensure that both software packages are up to date. If the mismatch between the automation software and the microscopy software remains, update the microscopy control command in the source code.

## Resource availability

### Lead contact

Further information and requests for resources and reagents should be directed to the lead contact, Rifka Vlijm (r.vlijm@rug.nl).

### Technical contact

Technical questions on executing this protocol should be directed to the technical contacts, Frank N. Mol (frank.mol@rug.nl) and Eline M.F. de Lange (e.m.f.de.lange@rug.nl).

### Materials availability

Plasmids generated in de Lange et al.^1^ are available upon request.

### Data and code availability

• All microscopy data reported in this paper have been deposited at DataverseNL and are publicly available at https://doi.org/10.34894/FNUHXH.
• All original code has been deposited at Zenodo and is publicly available at https://doi.org/10.5281/zenodo.11047200.
• Any additional information required to reanalyze the data reported in this paper is available from the lead contact upon request.

## Acknowledgments

This work was supported by NWO, the national research council of the Netherlands (grant number OCENW.M.21.106 to R.V.).

## Author contributions

Writing – original draft, F.N.M. and E.M.F.d.L.; writing – review and editing, F.N.M., E.M.F.d.L., I.J.v.d.K., and R.V.; supervision, I.J.v.d.K. and R.V.; funding acquisition, R.V.

## Declaration of interests

The authors declare no competing interests.
